# Light or Deep Pressure: Medical Staff Members Differ Extensively in Their Tactile Stimulation During Preterm Apnea

**DOI:** 10.3389/fped.2020.00102

**Published:** 2020-03-17

**Authors:** Sven Martin, Ulrich Herbert Thome, Martin Grunwald, Stephanie Margarete Mueller

**Affiliations:** ^1^Haptic Research Lab, Paul Flechsig Institute for Brain Research, Leipzig University, Leipzig, Germany; ^2^Department of Neonatology, University Hospital Leipzig, Leipzig, Germany

**Keywords:** neonatology, very low birth weight, pressure sensor, stimulation frequency, apnea of prematurity, treatment

## Abstract

**Background:** Even though tactile stimulation is common practice to terminate preterm apnea, the style and intensity of these interventions is not specified during theoretical or practical training and has never been clinically evaluated.

**Objective:** The present study was designed to analyze the various modes of tactile stimulation used to terminate preterm apnea and measure the pressure intensity and frequency of these stimulations.

**Methods:** A model with the size and weight of an actual preterm infant was equipped with sensor technology to measure stimulation pressure and frequency of tactile stimulation. Additionally a camera system was used to record hand positions and stimulation modes. Seventy medical staff members took part in the experiment.

**Results:** We found extreme between subjects differences in stimulation pressure that could not be explained by professional experience but, to a degree, depended on apnea intensity. Pressures ranged from 11.11 to 226.87 mbar during low intensity apnea and from 9.89 to 428.15 mbar during high intensity apnea. The majority of participants used rhythmic stimulation movements with a mean frequency of ~1 Hz. Different modes (rubbing, squeezing, tickling, and tapping) and finger positions were used.

**Conclusion:** Medical staff members intuitively adjust their tactile stimulation pressure depending on the premature infants' apnea intensity. However, mean pressure values varied greatly between subjects, with similar pressure ranges for low and high intensity apnea. The question remains which pressure intensities are necessary or sufficient for the task. It is reasonable to assume that some stimulation types may be more effective in rapidly terminating an apneic event.

## Introduction

Despite preventive measures, repetitive apneas occur in nearly all very low birth weight infants (1–4). Generally, the monitoring system will set off an alarm if an infant's oxygen saturation is low or bradycardia occurs which will prompt medical staff to investigate the cause of the alarm. If a central apnea is detected, the most established nonpharmacological practice is to administer gentle tactile stimulation to the infant's foot, hand, or torso. In most cases, these stimuli are sufficient to stabilize autonomous respiration. If gentle stimulation does not improve the parameters, more forceful tactile stimulations are applied, most commonly to the sole of the foot. Tactile stimulation has been shown to positively influence the occurrence and duration of preterm apnea (5–7).

While tactile stimulation is common practice, the style and intensity of tactile interventions have not been specified. Even though this intervention is used to treat highly critical situations with potentially life-long adverse effects if left untreated, the treatment approach is highly subjective. That means it is unclear what techniques and pressures are used by medical staff and if they differ in effectivity. We presume that each medical staff member has a different internal concept about what are gentle and what are strong tactile stimulations. To date no attempt has been made to objectively measure the different pressure intensities that are used to treat central apnea in premature infants. Similarly, no classification exists of the different modes of tactile foot stimulation. Do all staff members implicitly share an understanding of what ‘tactile stimulation‘ means? Or are different approaches like squeezing, rubbing or stroking applied? And if so, how do they choose one or the other and do they differ in pressure intensity?

In the present study, we want to document the various modes of stimulation and measure their corresponding frequencies and pressure intensities. We expected to find a significant association of pressure and frequency with apnea intensity. Also we expected to find differences in applied pressure between the various modes of stimulation (e.g., squeezing, rubbing, stroking). In addition, we intended to analyze if mode and intensity of tactile stimulation are influenced by professional experience and age. Due to subjective nature of the task we expected to find pronounced between-subjects variance.

For medical as well as ethical reasons, using prototype electrical sensors on a premature infant's body was not considered. Therefore we developed a model, which resembles the size and weight of an actual preterm infant. The model was equipped with sensor technology designed to measure stimulation pressure and frequency of tactile stimulation. Additionally a camera system was used to record hand positions and stimulation modes.

## Materials and Methods

### Model and Sensors

**Figure 1:**
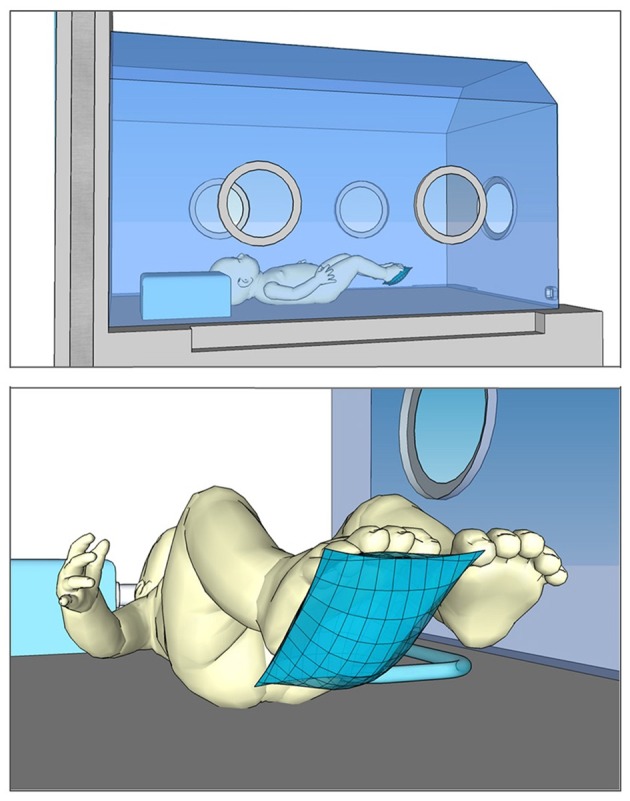
Schematic representation of the experimental setting. Top: Model preterm infant inside the incubator. Bottom: Water filled pouch (pressure sensor) on the model's foot.

We used a small baby puppet as a basis for our model. The hollow polyvinyl chloride (PVC) scaffolding of the doll was filled with small sandbags to match the weight of a premature infant of 1,000 g. A water filled pouch (31.1 mm × 38.2 mm × 9.1 mm) was attached to the sole of the right foot (Figure 1) which was connected to a pressure sensor (24PCCFA6D, Honeywell Int. Inc., Morristown, New Jersey, US) that registered any pressure that was applied to the pouch (measuring range: ±1034.21 mbar). The sensor output was registered by a Sigma/Delta analog-to-digital-converter (MCP3423-E/UN, Microchip Technology Inc., Chandler, Arizona, US) with a 14 Bit resolution and a mean sampling rate of 60 samples per second.

The infant model and measurement equipment were set up inside a state-of-the-art incubator. The manual stimulation process was videotaped via a webcam (Logitech C270; 640 × 480 pixel; 30 frames per second) which was attached to the incubator wall with a suction flange.

### Experimental Setting and Instructions

The study was conducted on several consecutive days on a quiet corridor of a university NICU. Upon arrival participants were informed about the setup and the experimental procedure. They were allowed to reach inside the incubator and familiarize themselves with the model infant and the sensory equipment. Demographic characteristics (age, gender, professional experience, work place, and handedness) of the participant were gathered. If no further questions occurred, the participant was asked to perform an apnea intervention as he/she would in real life if an apnea alarm of low urgency occurred. Low apnea urgency was defined by verbal instruction as a minimal drop in oxygen saturation (The alarm of the monitoring system of the patient indicates a drop of oxygen saturation slightly below the lower alarm limit (“yellow” alarm). After ~20 seconds of stimulation the participant was informed that the vital parameters of the infant model continued to deteriorate. Deep apnea was defined as prolonged apnea with very low oxygen saturation and bradycardia (“You realize that your present intervention is not effective in stopping the apnea. The oxygen saturation drops further and heart rate slows down. How would you proceed?”). To simulate a nearly natural course of events apnea intensity was not randomized. In a preliminary round nursing staff have been asked about their usual procedure. The answers were condensed into this standardized instruction.

The participants were free to use any mode and duration of stimulation they chose. Most participants indicated how they would assess the apnea before they began an intervention. Besides foot stimulation participants also chose to stimulate the hand or torso and lift up the head or upper body of the model. Participants did not wear surgical gloves during the experiment to avoid measurement distortion due to friction between the latex gloves and the pressure sensor.

The duration of the trial was between 5 and 10 min per participant.

The study was conducted in accordance with the World Medical Association Declaration of Helsinki and approved by the Institutional Review Board of the Medical Faculty, University of Leipzig.

### Parameters

Per person, two mean pressure values (mbar) were calculated from the raw data: one for low apnea intensity and one for high apnea intensity. Maximal pressure values were extracted for both apnea intensities. Furthermore, for those participants who performed rhythmic stimulation the stimulation frequency was computed.

To accomplish this, proprietary software was developed to synchronize the video signal and the data from the pressure sensor. Through this software we were able to denote the beginning and the end of the stimulation process and to exclude all pressure values ≤ 0 (overshooting or stimulation pauses) from the calculation of the means.

In addition, the video data were used to classify the positions of hand and fingers during foot stimulation (stimulation modes).

### Participants

*N* = 70 full-time nurses of a university NICU participated in the present study. Of these *n* = 42 worked at the Intensive Care Unit (ICU) and *n* = 28 were employed at Intermediate Care (IMC). Preterm infants on the IMC are generally more stable and apneic events occur less often, however, nurses take care of more infants simultaneously.

Mean age of the participants was *M* = 37.12 years (*SD* = 11.74; range: 19–60). Gender was predominantly female with only *n* = 3 male participants. Their mean professional experience was *M* = 14.03 years (*SD* = 12.93; range: 1–42). All participants were right handed according to a test of handedness (8), took part voluntarily and gave written informed consent.

All tests were conducted between 12:30 pm and 3 pm during shift changeover.

### Analyses

Due to limitations in normal distribution Wilcoxon signed rank and Mann Whitney *U*-tests were used for group comparisons. Spearman correlations (2-tailed) were used to assess the association between stimulation pressure, age and experience. Alpha was set at 5%. SPSS software version 24.0 was used (9). All datasets for this study are included in the manuscript/Supplementary Material.

## Results

### Apnea Intensity

Mean pressure during low intensity apnea (LIA) was *M* = 65.14 mbar (*SD* = 44.15; Median = 50.83; Range: 11.11–226.87) with an average maximal value of *M*(max_LIA) = 143.23 mbar (*SD* = 96.26; Median = 110.06; smallest maximal value = 16.93, largest maximal value = 456.70).

Mean pressure during high intensity apnea (HIA) was *M* = 126.09mbar (*SD* = 75.33; Median = 108.41; Range: 9.89 – 428.15) with an average maximal value of *M*(max_HIA) = 253.42mbar (SD = 147.76; Median = 240.01; smallest maximal value = 40.66, largest maximal value = 768.76).

Group medians (*z* = −6.781, *p* < 0.001) and the average maximal values (*z* = −6.463, *p* < 0.001) differed significantly between LIA and HIA.

Rhythmic stimulation was used by *n* = 62 participants during LIA and *n* = 58 participants during HIA. The remaining *n* = 9 (LIA) and *n* = 12 (HIA) participants performed single or multiple isolated stimulations. The frequency of rhythmic stimulation was statistically equal (*z* = −0.508, *p* = 0.611) for LIA (*M*_freq_=1.01 Hz; *SD* = 0.85; Median: 0.78; Range: 0.11–5.66) and HIA (*M*_freq_ = 0.92Hz; *SD* = 0.61; Median: 0.77; Range: 0.26–3.75).

**Figure 2:**
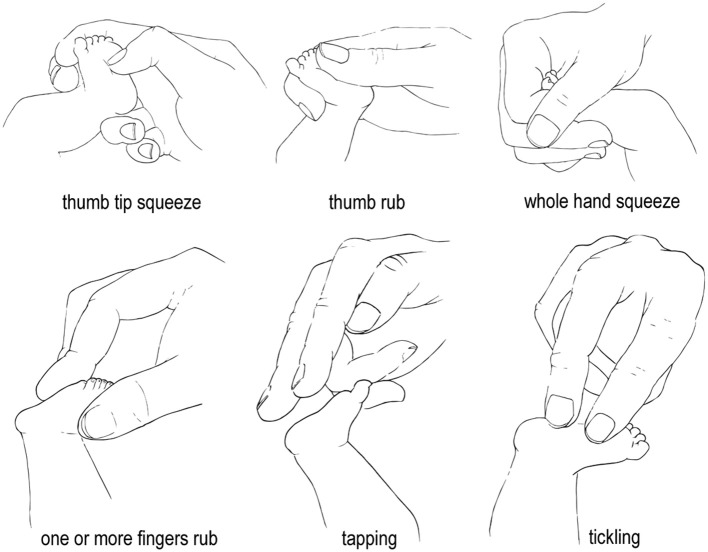
Illustration of typical finger positions and stimulation modes. Drawings by Anna Zender.

**Figure 3:**
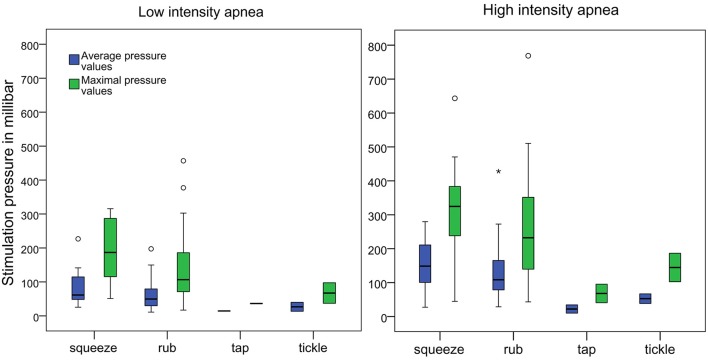
Boxplots of the average and maximal pressure values of the four stimulation types during low intensity apnea (LIA) and high intensity apnea (HIA). Fat horizontal lines mark the medians. Circles and stars indicate outlier values.

Two main modes of stimulation were observed: Rubbing movements were performed by 81.4 and 80% of participants during LIA and HIA, respectively. During both LIA and HIA 14.3% squeezed the foot. The remaining 4.3% (LIA) and 5.7% (HIA) participants performed tickle or tapping stimulations (Table 1). Pressure was statistically equal for rubbing and squeezing stimulation (Supplementary Table 1). To rub or squeeze the foot participants used six different finger positions (Figure 2). Most participants (*n* = 61) used the same mode of stimulation during LIA and HIA. Of these, *n* = 14 switched to a different finger. Mean pressure values varied greatly between subjects and fingers, ranging from 11.11 to 226.87 mbar during LIA and from 9.89 to 428.15 mbar during HIA (Figure 3).

**Table 1:** Mean and maximal stimulation pressure in millibar of different stimulation modes and their occurrence rates.

	**Squeezing**					**Rubbing**						**Tickle**	**Tapping**
	**Thumb**	**Thumb tip**	**Index finger**	**Two fingers**	**Whole hand**	**Thumb**	**Thumb tip**	**Index finger**	**Index finger tip**	**Two fingers**	**Whole hand**		
**Low Apnea Intensity (LIA)**													
	*N* = 1	*N* = 3	*N* = 1	*N* = 2	*N* = 3	*N* = 29	*N* = 10	*N* = 9	*N* = 3	*N* = 5	*N* = 1	*N* = 2	*N* = 1
Median	226.87	48,45	98.54	99.60	59.54	48.15	53.00	58.52	27.56	101.20	38.83	26.70	14.44
M	226.87	52.69	98.54	99.60	66.63	60.26	60.62	65.35	40.97	101.22	38.83	26.70	14.44
*SD*	-	9.28	-	59.18	44.99	37.86	39.26	37.42	23.44	60.47	-	18.66	
Median max	315.64	115,63	158.44	248.74	163.12	94.52	99.84	106.73	64.83	264.85	120.80	67.21	36.30
M max	315.64	168.20	158.44	248.74	155.57	125.47	129.43	152.75	94.33	240.13	120.80	67.21	36.30
*SD* max	-	114.72	-	53.97	100.76	84.18	78.16	109.99	54.97	148.02	-	42.89	
**High Apnea Intensity (HIA)**													
	*N* = 2	*N* = 3	*N* = 0	*N* = 0	*N* = 5	*N* = 27	*N* = 12	*N* = 8	*N* = 2	*N* = 6	*N* = 1	*N* = 2	*N* = 2
Median	127.07	121.52	-	-	175.59	87.77	123.19	122.78	158.04	122.39	143.15	52.63	22.11
M	127.07	134.63	-	-	176.30	119.54	125.49	142.86	158.04	133.59	143.15	52.63	22.11
*SD*	141.13	53.17	-	-	73.20	87.92	45.28	83.57	73.82	54.01	-	19.93	17.28
Median max	180.15	238.15	-	-	339.25	155.57	279.58	203.67	350.12	288.67	354.08	144.56	68.01
M max	180.15	273.78	-	-	407.72	222.79	262.62	252.44	350.12	308.89	354.08	144.56	68.01
*SD* max	191.61	96.69	-	-	153.12	155.07	122.35	143.87	219.17	120.22	-	59.25	38.69

*M, mean pressure across participants; SD, standard deviation; Mmax, mean of the maximal pressure values; SDmax, standard deviation of Mmax*.

### Professional Experience, Age and Workplace

Stimulation pressure of the participants who worked at the ICU did not differ from those who worked at the IMC (Supplementary Table 2).

Correlative analyses of age and stimulation pressure (mean and maximal value) did not reveal any associations (LIA: *r*_mean_ =0.019, *p* = 0.873; *r*_max_ = 0.135, *p* = 0.266; HIA: *r*_mean_ = 0.049, *p* =0.689; *r*_max_ =0.157, *p* = 0.195). Professional experience and stimulation pressure did not show any correlative associations either (LIA: *r*_mean_ = – 0.039, *p* = 0.749; *r*_max_ =0.071, *p* = 0.559; HIA: *r*_mean_ =0.062, *p* = 0.610; *r*_max_ =0.159, *p* = 0.188).

We did find, however, highly significant correlation coefficients of the pressures applied during LIA and HIA. The mean pressures used during LIA and HIA were strongly correlated (*r*_mean_ =0.689, *p* < 0.001). The maximal pressure values used during LIA and HIA were also strongly correlated (*r*_max_ =0.683, *p* < 0.001).

## Discussion

The present study was designed to analyze the various modes of tactile stimulation used to terminate preterm apnea and measure the pressure intensity and frequency of these stimulations on a model puppet. Even though tactile stimulation is common practice to terminate apneic events, the style and intensity of these interventions is not specified during theoretical or practical training and has never been evaluated.

As hypothesized, we found significant differences in stimulation pressures depending on apnea intensity. Participants applied significantly less pressure when heart rate and oxygen level were borderline normal than when heart rate and oxygen level continued to decline. Accordingly, medical staff members intuitively adjust their tactile stimulation pressure depending on the premature infants' biomarkers. However, mean pressure values varied greatly between subjects, ranging from 11.11 to 226.87mbar during low intensity apnea and from 9.89 to 428.15mbar during high intensity apnea. That means that during both low and high apnea intensity participants showed a similarly wide range of stimulation pressures. We also found a highly significant correlation of pressures used during LIA and HIA. In other words, participants who used strong pressure during LIA were also among those who used strong pressure during HIA. Therefore, the question arises, if there is a minimally necessary pressure to influence an apneic event and if some of the stronger pressure values may be excessive. The absolutely largest maximal pressure value applied momentarily by a participant was 768.76 mbar. Given the very delicate nature of premature infants' skin some of the shear forces, especially if applied with a fingernail, may be unnecessarily painful, possibly even damaging.

To offer some reference to the applied pressures we conducted a comparative measurement with a conventional blood pressure gauge for preterm infants. As a result, the maximal pressure during blood pressure measurement was 95.79 mbar. In relation to this, only the mean tactile pressure during low intensity apnea (M_LIA_= 65.14 mbar) was smaller than the maximal value of the blood pressure cuff. During high intensity apnea all but 4 participants used mean pressures that were stronger than the maximal values during blood pressure reading.

As expected, medical staff members used different modes (rubbing, squeezing, tickling, and tapping) and finger positions to perform foot stimulations. Rubbing and squeezing were most commonly used, but did not differ in stimulation pressure due to high levels of variance. Descriptively tickling and tapping reached the lowest mean pressure values. Overall six different finger positions were observed. Statistical comparisons of the mean pressures of different finger positions were not possible due to low numbers. The majority of participants used rhythmic stimulation movements with a mean frequency of ~1 Hz. Stimulation frequency was the same during low and high intensity apnea.

The variance in mode and force of stimulation was not associated with professional experience (range: 1–42 years) or workplace (ICU or IMC). We conclude, that the between subjects variance in stimulation pressure cannot be explained by experience-based learning.

Because we used a model instead of a real infant and the biological parameters of the child were announced instead of indicated by an alarm the results of the present study should be used with caution. Even though the experimenter repeatedly urged the participants to do as they would in real life, the artificial situation may have influence the applied pressures.

Since all medical staff members showed the same confidence in their ability to disrupt an apneic event with their individual stimulation strategy, the question remains which pressure intensities are necessary or sufficient for the task. It is reasonable to assume that some stimulation types may be more effective in rapidly terminating an apneic event.

Future studies should also try to assess whether different modes of stimulation and pressure intensities influence the duration and occurrence frequency of apnea.

## Data Availability Statement

All datasets generated for this study are included in the article/Supplementary Material.

## Ethics Statement

The studies involving human participants were reviewed and approved by Institutional Review Board of the Medical Faculty, University of Leipzig. The patients/participants provided their written informed consent to participate in this study.

## Author Contributions

SMa organized the database. SMu performed the statistical analysis. SMa wrote the first draft of the manuscript. All authors contributed to conception, design of the study, manuscript revision, read, and approved the submitted version.

### Conflict of Interest

The authors declare that the research was conducted in the absence of any commercial or financial relationships that could be construed as a potential conflict of interest.
