# Supplementary material for: Light or Deep Pressure: Medical Staff Members Differ Extensively in Their Tactile Stimulation During Preterm Apnea
Source: Front Pediatr. 2020 Mar 17;8:102. doi: 10.3389/fped.2020.00102 (PMC7089873; doi:10.3389/fped.2020.00102)
Supplement: Supplementary file 1 [file Table_1.docx]

Supplementary Table 1. Comparison of the applied pressure in millibar during rubbing and squeezing stimulation.

|  | Stimulation mode | | |  |
| --- | --- | --- | --- | --- |
| apnea intensity |  | squeezing | rubbing | *p*-value |
| LIA | *Median* | 59.54 | 49.63 | .165 |
|  | *M (SD)* | 85.31 (62.96) | 63.32 (40.22) |  |
|  | *Median_max* | 163.12 | 106.73 | .051 |
|  | *M*max *(SD)* | 187.84 (96.35) | 138.81 (95.36) |  |
| HIA | *Median* | 121.52 | 111.30 | .186 |
|  | *M (SD)* | 145.85 (75.96) | 129.64 (73.72) |  |
|  | *Median_max* | 333.31 | 244.18 | .180 |
|  | *M*max *(SD)* | 322.73 (170.70) | 255.80 (143.73) |  |

LIA: low intensity apnea; HIA: high intensity apnea; *M* = mean pressure across participants; *SD* = standard deviation; *M*max = mean of the maximal pressure values; Mann-Whitney-U-Test.
