# Supplementary material for: Light or Deep Pressure: Medical Staff Members Differ Extensively in Their Tactile Stimulation During Preterm Apnea
Source: Front Pediatr. 2020 Mar 17;8:102. doi: 10.3389/fped.2020.00102 (PMC7089873; doi:10.3389/fped.2020.00102)
Supplement: Supplementary file 2 [file Table_2.docx]

Supplementary Table 2. Group comparison: Mean and maximal stimulation pressure in millibar of ICU and IMC participants.

| apnea intensity |  | ICU | IMC | *p-*value |
| --- | --- | --- | --- | --- |
| LIA | *Median* | 48.45 | 50.83 | .388 |
|  | *M (SD)* | 70.07 (50.22) | 56.17 (32.16) |  |
|  | *Median_max* | 111.93 | 107.46 | .581 |
|  | *Mmax (SD)* | 148.87 (102.17) | 131.07 (87,01) |  |
| HIA | *Median* | 117.72 | 87.77 | .172 |
|  | *M (SD)* | 133.87 (67.54) | 115.12 (85.02) |  |
|  | *Median_max* | 265.42 | 186.46 | .494 |
|  | *M*max *(SD)* | 261.80 (138.11) | 247.08 (166.55) |  |

ICU: Intensive Care Unit; IMC: Intermediate Care; LIA: low intensity apnea; HIA: high intensity apnea; *M* = mean pressure across participants; *SD* = standard deviation; *M*max = mean of the maximal pressure values; Mann-Whitney-U-Test.
